# Reuse, remanufacturing and recycling in the steel sector

**DOI:** 10.1098/rsta.2023.0244

**Published:** 2024-11-04

**Authors:** C. Davis, R. Hall, S. Hazra, K. Debattista, S. Zhuang, J. Duan, Z. Li, J. Shenton, D. Panni, A. Halfpenny

**Affiliations:** ^1^ WMG, University of Warwick, Coventry CV4 7AL, UK; ^2^ School of Engineering, Newcastle University, Newcastle upon Tyne NE1 7RU, UK; ^3^ JCB, Rocester, Staffordshire ST14 5JP, UK; ^4^ Hottinger Bruel & Kjaer UK Ltd., Advanced Manufacturing Park Technology Centre, Brunel Way, Catcliffe, Rotherham, South Yorkshire S60 5WG, UK

**Keywords:** steel, reuse, remanufacturing, recycling

## Abstract

The global steel sector is undergoing a transition from being a major CO_2_ emitter to a more sustainable circular material service provider, moving towards (near) net zero CO_2_ through combined strategies of reuse, remanufacturing, recycling and changes to primary steelmaking. This paper considers the transition using the UK as an example, based on the current sector state and future plans/opportunities. Some key enablers/barriers have been identified, and case studies are presented on the current state of knowledge and technology developments. For example, increasing reuse/remanufacturing requires data on the component’s remaining life at the end-of-product life; in this work use of in-service monitoring for steel-intensive applications in the transport sector is discussed identifying sensor types/locations for fatigue loading assessment for different use conditions to feed into material/product passports for reuse/remanufacturing decisions. Increased recycling of obsolete scrap has implications for composition control with increases in residual elements, such as Cu, Sn, Cr and Ni inevitable. Current and future approaches to recycling and scrap sorting are discussed along with case studies for how residual elements affect microstructural development during steel processing, including effects on recrystallization, phase transformation and fine-scale precipitation, which potentially could be exploited to give increases in product strength.

This article is part of the discussion meeting issue ‘Sustainable metals: science and systems’.

## 1. Approaches to decarbonize the steel sector

Steel is fundamental for modern life; it is the most used engineering material in the world and is an essential material for the transition to net zero through its use in the energy and construction sectors. In 2020, global steel production was 1860 Mt, and each tonne of steel produced led to the emission of on average 1.85 t of CO_2_ into the atmosphere; total global emissions from steel production were estimated at around 2.6 Bnt of CO_2_, representing about 8% of total global emissions [1]. There is a clear need to reduce the carbon footprint of the steel sector through the adoption of alternative steelmaking technologies, decarbonizing current steelmaking and considering approaches for material efficiency to decrease the amount of ‘new’ steel required.

Globally a shift in steelmaking technologies is occurring: many manufacturers are moving away from the high CO_2_-emitting traditional blast furnace-basic oxygen furnace (BF-BOF) route to the lower CO_2_-emitting electric arc furnace (EAF) steelmaking using scrap steel as a feedstock to charge the EAF and direct reduced iron (DRI) for dilution [2]. This is particularly evident in Europe and North America where there are significant scrap steel volumes that can be used as feedstock. It should be noted that limited global scrap steel supply and continuously increasing demand for steel means that BF-BOF production will need to continue at least in the short-medium term. DRI is the reduction of iron ore in a solid state, while hydrogen DRI (H-DRI) is a promising emerging technology for low-emission steelmaking where the process outputs are iron and steam. H-DRI is not a full substitute for scrap in the EAF steelmaking process, rather it is generally used to supplement and enhance the scrap charge [3]. EAF steel production using scrap reduces CO_2_ emissions by around 1.5 tCO_2_/t of new steel manufactured, EAF steelmaking combined with H-DRI has the potential to reduce emissions to as low as 0.1 tCO_2_/t of new steel manufactured if using green hydrogen [1,4]. Other alternative steelmaking technologies with low CO_2_ emissions, such as the molten oxide electrolysis, are also being piloted [5], along with carbon capture use and storage applied to the steel sector [6].

In addition to the decarbonization of steel production, material efficiency approaches can be adopted to reduce the amount of ‘new’ steel required. The well-established major strategies for reducing material demand include the design of longer-lasting products, modularization and remanufacturing, component reuse and designing products with less material [7]. Various barriers, including economic, regulatory and social factors, have been identified that need to be overcome to increase material efficiency. The majority of steel components are underexploited because the steel retains its functionality when the product is discarded [8]. For products with no, or an unchanging, use phase energy requirement, remanufacturing (or reuse) can save energy, whereas for typically powered products, small changes in use phase efficiency are more significant than energy savings from materials production and manufacturing [9]. Gutowski *et al*. [9] highlighted the dominance of use phase emissions of powered products, and their conclusions generally apply to changes in the efficiency of mature, stable technologies. However, the current move to electrification in the automotive sector means that there is a step change in in-service emissions; therefore, despite the risk of the lower relative efficiency of a remanufactured product, its *lower purchase price* [9] will encourage quicker adoption of a cleaner technology that subsequently hastens the reduction of overall emissions within a society. Increasing reuse and remanufacturing at the product or component level requires consideration of the energy efficiency for both the manufacturing and use phases to ensure appropriate decisions are made on remanufacturing or potential component reuse in updated products. Additional barriers identified for reuse and remanufacturing include undeveloped supply chains and incomplete knowledge of the material state (potential need for recertification [7]) with material (product) passports being proposed to support circularity and sustainability sector [10].

In this paper, the route towards near net zero for the UK steel sector is discussed considering the announced plans for changes in steelmaking technologies and potential increases in steel reuse and remanufacturing. Case studies are presented that focus on specific technology barriers to sustainability in the UK steel sector: increased scrap use to support the transition to EAF production, considering scrap sortation and the implications of higher residual element content in the steel composition on microstructure development during processing; and assessment of a component’s remaining life at the end-of-product life (using sensors for fatigue loading assessment for different use conditions) in the heavy goods sector for input into material/product passports.

## 2. Introduction to route to (near) net zero in the UK

The United Kingdom produces around 7 Mt of crude steel each year, with associated CO_2_ emissions of approximately 13.2 Mt; to give this context, it is 2% of total UK territorial emissions and 14% of UK industrial greenhouse gas emissions [11]. Currently, UK steelmaking uses a mix of BF-BOF and EAF steelmaking in a ratio of 81:19%, respectively, as of 2023. Two of the UK’s steelmakers, Tata Steel UK and British Steel, have recently announced that they are moving to EAF steelmaking in the near future showing that the UK steel industry is refocusing on lower emission steelmaking routes [12,13].

**Figure 1.**
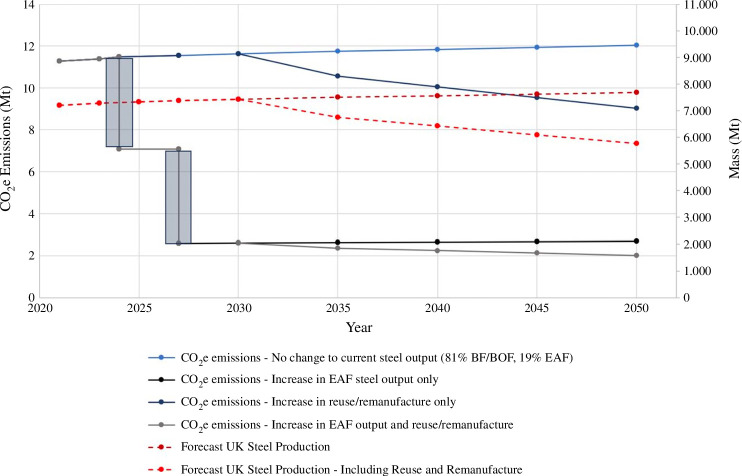
UK steel production emission predictions for different scenarios, where dashed lines are UK steel production and solid lines are CO_2_e emissions. The shaded boxes represent uncertainty on timeline for BF/BOF phase-out, which are based on press releases [6,7].

Modelling shows the potential impact that increased steel circularity—reuse, remanufacture and EAF-based steel recycling—could have on UK steel industry emissions. Table 1 shows different emission modelling scenarios from 2021 to 2050, with the inclusion of EAF steelmaking at British Steel in 2025 and Tata Steel UK in 2027, based on recently announced projected timelines. Figure 1 shows the projected emissions reductions based on the following assumptions:

— Steel production volumes in the UK continue to grow in proportion with the UK population. The Office for National Statistics data predicts the UK population will increase beyond 71 M by 2050, and data from WorldSteel reports that each person in the UK consumes 0.161 tpa of steel [14,15]. It is assumed that manufacturers in the UK will continue to design products using current design principles. Reduction of steel use, through either simply using less or by more efficient use of steel (e.g. lower volumes of higher strength steels replacing greater volumes of lower strength steels), would have a direct impact on estimated CO_2_ emissions (CO_2_e). The extent and temporality of this impact is debated; however, with emissions reduction impacts ranging from 12 to 38%, the timeliness of design change adoption is also unknown [16]. Similarly increased yield from steel production would have a positive effect on steelmaking CO_2_e emissions, EAF steelmaking has been shown to be roughly twice as resource-efficient as BF-BOF steel production [17].
— EAF steelmaking will fully replace BF-BOF production by 2027, and the transition will be completed in discrete step changes where there is no overlap between EAF and BF-BOF at either British Steel or Tata Steel UK sites.
— The amount of steel produced in the UK does not change when the switch from BF-BOF to EAF occurs (i.e. similar product mix is achieved).
— For the modelling, it has been assumed that EAF steelmaking is supported by fully renewable, green energy supply. It is appreciated that this is not the current case and that it is unlikely to be the full case by 2050.
— As it is not currently possible to manufacture all grades of steel from recycled scrap steel in an EAF, it is expected that a pure iron source will be required for the dilution of impurities. Data for different DRI types or uses are not shown in the emissions models as there are currently no published plans for use of DRI by UK steelmakers. Studies into different DRI steelmaking routes give estimates for embodied emissions ranging from 0.1 tCO_2_e/t new steel for H-DRI to 0.3 tCO_2_e/t new steel for biosyngas DRI [11,17].
— UK manufacturing is becoming increasingly engaged with a circular economy. A report by the Green Alliance suggests that material consumption in the construction industry could be reduced by as much as 35% by 2035, including hot rolled and strip steel products [18]. The automotive industry is also pushing towards using circular principles of lifetime extension, reuse and remanufacture. As an example, the Renault Group launched Europe’s first circular vehicle factory in 2021 [19–22]. It is not possible to know the exact impact or extent of the circular economy on steel use in UK manufacturing. To show the potential impact on emissions, it has been assumed that in 2035 10% of UK-made steel will be reused or remanufactured and that there will be a steady increase to 25% by 2050.

**Table 1.** Emissions modelling scenarios for future steel production in the UK.

year	2021	2023	2025	2027	2030	2035	2040	2045	2050
forecast UK steel production based on population growth only (Mt)	7.20	7.27	7.33	7.37	7.42	7.50	7.56	7.62	7.68
BF-BOF/EAF production ratio (%)	81:19	81:19	41:59	0:100	0:100	0:100	0:100	0:100	0:100
steel in reuse and remanufacture (percentage mass produced)	0	0	0	0	0	10	15	20	25
forecast UK steel production including reuse and remanufacture (Mt)	7.20	7.27	7.33	7.37	7.42	6.75	6.42	6.10	5.76

Figure 1 shows that the biggest impact on steel production CO_2_e emissions comes from the shift from BF-BOF production to EAF steelmaking. Projections show that in 2050 CO_2_e emissions would be 12.03 Mtpa with no change from the current BF-BOF production mix, whereas this could be as low as 2.70 Mtpa with only EAF production and no reuse or remanufacture, based on the assumptions given. The effect on emissions is clear with direct step-change reductions as the switch to EAFs occurs. Transitioning to EAF production relies on there being enough scrap steel to support UK manufacture. Currently, the UK produces approximately 11.3 Mtpa of scrap steel, the majority of which (approx. 80%) is exported. Therefore, in principle, there is sufficient scrap steel as feedstock for the current UK primary steel production needs, although consideration of the supply chains and quality control is required and discussed later in §4. Section 4 includes consideration of scrap composition, specifications, sortation and therefore impact on the residual element content in steels produced through the high scrap route, such as via EAF. Case studies for the implications of higher residual element compositions on processing and property development in steel are given illustrating the areas that need to be considered by producers when manufacturing these steels.

Figure 1 also illustrates the potential impact of reuse and remanufacture on CO_2_e emissions, which for the UK is a minor factor compared with changes in steelmaking technologies. The model shows that in 2050, with fully EAF steel production and 25% reuse and remanufacture, CO_2_e emissions would be 2.08 Mtpa. However, in the long term, reuse and remanufacture and other material efficiency approaches are also important in achieving a near net zero and sustainable steel sector. This is discussed in more depth in §3, where the case study focuses on the assessment of material condition at end of life (EoL) for input into material/product passports as one of the technical barriers to greater reuse and remanufacture [23].

## 3. Reuse and remanufacture

Reuse is to use a component or product again at the end of its life, which may require it to be repaired, refurbished or remanufactured (including repurposing of a component into another product). Remanufacturing is the process of returning a used product to at least the original performance specification with a warranty that is at least equal to that of a newly manufactured equivalent [24]. Reuse extends the lifetime of products, therefore reducing the amount of primary-produced materials, such as steel, required. Repair for life extension and reuse is already common (for example, regular servicing of cars and second-hand market) and can be accounted for in the nominal lifetime of the product. Remanufacture, where components may be reused in different products, is a less mature approach but a developing area. In the construction sector, modular design and demountable connections allow steel components such as I-beams to be reused or remanufactured.

Table 1 and figure 1 are based on a current assumption of minimal reuse for steels, i.e. not separating out current practices of life extension (repair/refurbishment) and second-hand markets. An increased focus on reuse as part of a circular economy is expected to reduce primary material demand and a review of the current approaches to (steel) reuse and remanufacturing in the key UK sectors of transport and construction are discussed below.

### (a) The UK transport sector

The UK aspires that by 2050 transport will have zero emissions at the point of use and the materials for manufacture will operate within a circular economy framework [25]. The circular economy framework includes reuse (repair, refurbishment and remanufacturing) and recycling. The Energy Transition Commission, a non-governmental organization, estimates that by 2050, the circular economy has the potential to reduce emissions by 40% globally and 56% within Europe [26]. The UK’s automotive remanufacturing sector is estimated to be worth £2.4 bn in 2021 [27]. Components that are frequently remanufactured include starter motors, alternators, transmissions and engines. The majority of these components are recovered at Authorised Treatment Facilities, which are required by law to process EoL vehicles (in accordance with Directive 2000/53/EC). These are high-value components in a vehicle, but, from a steel sector perspective, they are low volume. Remanufacturing of body structures (high-volume steel components) is not currently done, with the steel being recycled. Remanufacturing and recycling of heavy-duty off-road (HDOR) vehicles is not legislated and is a comparatively minor activity, but when remanufacturing is carried out, it is potentially profitable [28]. For example, a case study analysis was conducted for the manual disassembly of an 8-tonne forklift truck focusing on the 16 largest component assemblies such as its ‘motor and gearbox’ [29]. The profit margins from remanufacturing and reuse meant that the economic break-even point was to disassemble just 75% of the mass of the vehicle to remanufacture 10%, reuse 5% and recycle the remaining components. Comparing a newly manufactured HDOR vehicle with a remanufactured product, primary production results in 4.4 tCO_2_e, while remanufacturing at the OEM factory results in 1.3 tCO_2_e, giving a 3.1 tCO_2_e reduction (70%) in emissions [30]. Therefore, it appears that there is scope for increased remanufacturing and reuse in the HDOR sector.

### (b) The construction industry

The construction industry is implementing circular economy principles. For example, according to Singapore’s Ministry of Sustainability and Environment, Singapore recycles 99% of construction waste [31] where the Building and Construction Authority’s demolition protocol requires contractors to maximize the recovery of demolition materials [32]. The protocol was accompanied by legislation that acknowledged the reusability of steel sections and outlined a process for re-certifying the quality of post-demolition steel. In the UK, London will require projects that apply for planning permission to submit a circular economy statement to support their application [33]. An analysis of the cost and risks associated with the reuse of construction steel beams for the UK market has identified challenges associated with the supply chain for steel reuse [34]. A significant barrier is trust in the quality of used steel and the need to hold used steel for long periods of time because of the gap between supply and demand.

An important factor in improving the productivity of remanufacturing is prior knowledge of the quality of the product. In linear manufacturing, the quality of raw materials, components, sub-assembly and the product is defined at the product development stage through standards and commercial contracts. The quality of an EoL product (the ‘core’), however, will be variable because it strongly depends on the history of the product. As a result, the process of remanufacturing broadly requires the following steps: regular inspections and use history [35]: acquisition of the core, disassembly of the core for inspection, disposal of worn-out components, cleaning and further inspection before adding new, perhaps, upgraded components. Before delivery to the market, the product has to undergo final inspection and testing. Knowing the quality or condition of the EoL product prior to processing is key to the productivity of the process, which can be defined via the concept of ‘certainty of product quality’ [36]. Products that have been used are characterized by variable quality, and this quality can be difficult to value in a marketplace [37]. Characterizing the quality of an EoL product is therefore essential for avoiding the high costs of EoL processing and ensuring a supply of EoL products that can be processed. As discussed above, providing information on EoL quality requires materials passports containing data on the original product material and in-service history to determine effective damage (or life reduction) criteria to ensure appropriate value and properties can be assigned to the component for reuse/remanufacturing. A case study on how EoL data for materials passports can be acquired using low-cost sensors and new data analytic approaches for in-service monitoring for components in the HDOR sector is provided below.

#### (i) Case study on in-service monitoring for material data passports

**Figure 2.**
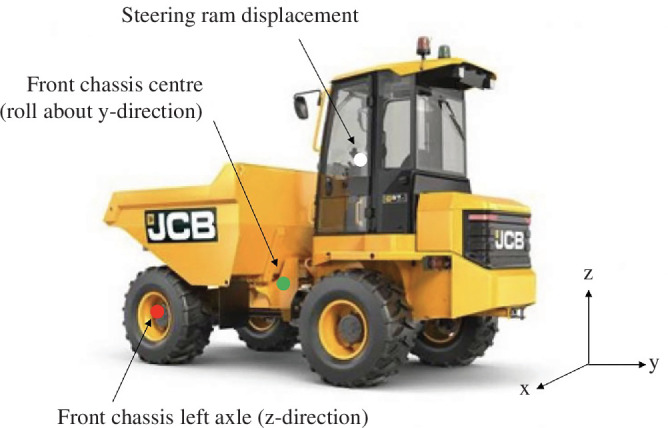
JCB site dumper vehicle with key sensor locations marked that provide sufficient data for assessment of vehicle loading.

It is known that the manner in which a product is used in-service has a dominant impact on its EoL quality [36]. A series of road trials were carried out on an HDOR vehicle to investigate techniques to identify how the vehicle was used in-service and how the information can be used to determine EoL quality. The study focused on HDOR vehicles because the UK is one of the largest manufacturers of HDOR vehicles in Europe [38]. Determining the in-service life for HDOR vehicles is challenging because they are designed to be flexible in the manner they can be operated; therefore, techniques used in health monitoring for vehicles for durability studies were investigated [39]. A JCB site dumper vehicle was instrumented with five accelerometers, six pressure sensors, two extensometers and two inertial measurement units (which measured acceleration along six degrees of freedom: three linear and three angular accelerations), meaning 25 degrees of freedom were measured. In the final assessment (discussed below), it was found that only three sensors were required to provide sufficient information to determine the loading on the system, as shown in figure 2.

The data from the sensors were collected using a combination of manual collections and JCB’s LiveLink ICT system, which is designed to collect sensor data remotely from vehicles in the field. The impact of in-service use was investigated by varying three factors: vehicle load, the terrain the vehicle is operated on and the experience of the operator. ‘Vehicle load’ and ‘Terrain’ were investigated because adding load on a vehicle and driving on rougher terrain are primary functions that increase the stresses on the vehicle chassis. ‘Operator experience’ was chosen because these vehicles are frequently leased during service, and operators with lower experience tend to make greater steering and power inputs, resulting in greater accelerations and stresses in the chassis. Each factor was varied to three levels. A full factorial experiment was carried out by driving the vehicle around a test track 27 times according to the test conditions. The data were analysed for average peak values and power spectrum density (PSD) from the accelerometers. The average peak values were analysed with analysis of variance (ANOVA) and the PSD using artificial intelligence (AI) models that classify patterns in spectral data [40]. Two-thirds of the data was used to train an AI model, and the remaining third was used to test the model.

**Figure 3.**
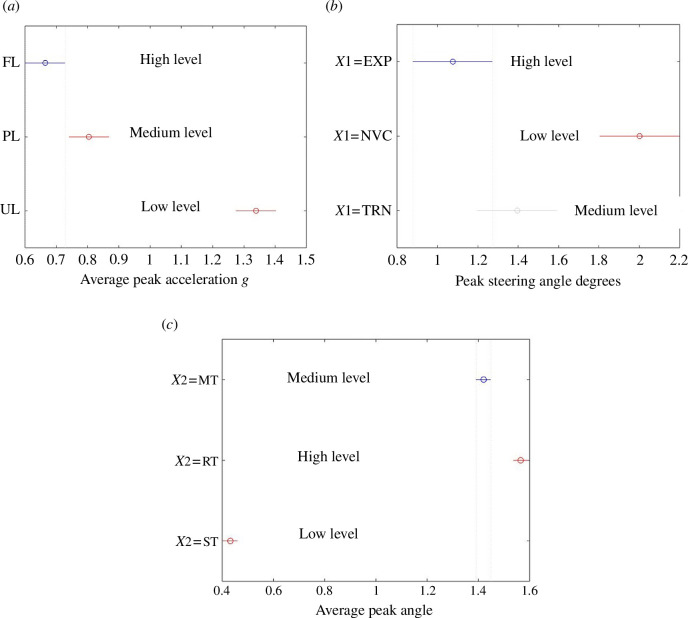
(*a*) Average peak acceleration for sensor ‘Front chassis left axle mount’ with variation in ‘Vehicle Load’ (FL: fully laden; PL: partially laden; UL: unladen). (*b*) Average peak angles for roll sensor of the ‘Front chassis centre’ IMU with variation in ‘Terrain’. (*c*) Average peak steering angles with variation in ‘Driver experience’.

The results of the ANOVA and AI analysis showed that:

1. ‘Vehicle load’ was predicted by the accelerometer on ‘Front chassis left axle mount’ to 95% confidence (figure 3*a*).
2. ‘Terrain’ was predicted by the roll sensor of the ‘Front chassis centre’ IMU to 95% confidence (figure 3*b*).
3. ‘Driver experience’ was predicted by steering angle data calculated from ‘Steering ram displacement’. ‘Novice’ drivers were distinguishable from ‘Trained’ and ‘Expert’ drivers with 95% confidence, but ‘Expert’ and ‘Trained’ drivers could only be distinguished with less than 95% confidence (figure 3*c*).
4. AI was only able to predict Terrain with 70% accuracy. The AI method could be improved by using alternative characterizations of the time-series data such as shock response spectrums that better capture peak loadings.

It was concluded that the in-service use of the HDOR vehicle analysed in this study can be identified by monitoring the peak values from three sensors positioned at key locations on the vehicle. For this class of vehicles, accelerometers, roll sensors and displacement sensors are increasingly common and can therefore provide data for estimating the in-service usage of the vehicle. The outputs from these sensors can then be used to determine the effective load cycles for the vehicles and hence fatigue damage. The significant new outcomes from this case study are that relatively few sensors are required to provide the necessary data for fatigue life assessment. Utilization of existing sensor data means that the majority of required investment is in the initial trials for baseline data and data processing to provide the EoL assessment framework. Fatigue damage, and total fatigue life, for different loading cycles can be determined in test facilities, and therefore the accumulated damage (as a percentage of total life) for an in-service vehicle throughout its service life is determined. Incorporation of the EoL quality into material passports for components will allow appropriate decisions to be made on the vehicle EoL for life extension or disassembly and component reuse, remanufacture or recycling. The extension of this approach to other transport applications requires investment in sensing and adoption of materials passports, along with consideration of ownership models and customer acceptance to facilitate the reuse, remanufacturing and upcycling of the EoL steel products.

## 4. Recycling

It was identified in §2 that there is a major transition to EAF steel production, which has the potential for low CO_2_ emissions through the use of high scrap steel content as feedstock. It is therefore important to consider the nature, quality and impact of scrap steel. Steel scrap can be generated from the steelmaking process (known as ‘internal scrap’), during the manufacturing of steel products (known as ‘pre-consumer scrap’), and at the end of steel products' useful life (known as ‘post-consumer scrap’). Steel scrap recycling primarily concerns the latter two types of scraps as the steelmakers consume their internal scrap effectively within their manufacturing process. Scrap availability depends on the average life and the volume of steel products. The average service life of steel products varies from a few weeks for steel packaging to up to 100 years for buildings and infrastructure, with the average lifespan of a steel product being 40 years [41]; by contrast, the average life of a passenger car in the UK is 13.5 years [42]. WorldSteel Association estimates suggest that global EoL ferrous scrap availability stood at about 400 Mt in 2019 and will reach about 600 Mt in 2030 and 900 Mt in 2050 [43]. Increased reuse and remanufacturing may alter the amount of scrap availability in the future.

Clearly, entirely scrap-based steel production is unlikely to meet the predicted global steel demand of 2.3 billion tonnes per year in 2050, and that is why the global steel industries are developing a broad portfolio of technological options to achieve the 2050 net zero target. However, upcycling steel scrap to high-quality steel products at competitive costs plays a critical role in achieving the 2050 net zero target for the steel industry considering the significantly increased availability of scrap and the impact of scrap usage in reducing CO_2_ emissions. As explained in §2, this is particularly the case for the UK steel industry as the UK produces over 11 Mt steel scrap per year, which could be more than sufficient in quantity for the production of 7–8 Mt per year crude steel. Increasing scrap usage is a strategy to help decarbonize the UK steel industry and maintain its sustainability considering the excessive scrap supply in the UK and the significantly reduced CO_2_ emissions of using recycled scrap in steelmaking. To achieve this, great efforts are being made and continuous efforts are required to address various challenges of steel scrap recycling from the scrap supply chain to technology development for improved quality, in addition to environmental/social impacts and policy interventions.

Efficiently getting the right volume and the required quality of steel scrap has always been a challenge to the steelmaker. The steelmakers purchase steel scrap from recyclers (scrap dealers) and/or obtain the scrap from their own scrap yard. The UK steel industry relies on scrap dealers for the scrap supply, while some global steelmakers are starting to intentionally increase their ownership of scrap yards for the security of scrap supply and scrap quality control [44]. The current UK steel scrap supply is formed by four tiers with tier 3 and tier 4 suppliers being based in small cities and towns collecting scrap from different sources and supplying them to tier 1 and tier 2 suppliers without much proper sorting. Tier 1 and tier 2 scrap dealers are capable of processing scraps in large volumes and dominate the scrap supply to the UK and overseas steelmakers. One of the main challenges for efficient scrap recycling is the differing motives regarding scrap quality, technical understanding, scrap volume, delivery and price between the steelmaker and the scrap dealer. Close collaboration between the steelmaker and scrap supplier is essential for efficiently upcycling the steel scrap. The other challenges could be converting the current sequential, linear scrap supply chain to a digitalized, human–machine interactive scrap supply chain to enable the transition to steel scrap recycling in a circular economy manner and the adoption of new supply chain practices such as a closed-loop supply chain.

The well-known challenge for converting scrap to high-quality steel products via EAF steelmaking is the scrap quality. The scrap supplied to the steelmaker (merchant scrap) in general is a mixed material without adequate chemistry measurement, which limits the accuracy, consistency and compatibility with the chemistry of the products to be manufactured. Several available steel scrap specifications such as the UK Ferrous Metals Specifications; EU-27 Steel Scrap Specification; Japanese standards for ferrous scrap; and ISRI Scrap Specifications do not have complete chemistry requirements. The UK steel scrap categories and expected residual element levels, as estimated from the EU standards, are given in table 2. It is difficult to determine the exact amount of each type of scrap category in the UK, but Spooner *et al*. [45] used a range of sources to estimate the scrap arising and determined that approximately 46.7% falls into categories 0A/1/2; 27.7% in category 3B; 10.4% in category 8A; 5.1% in categories 9A/9D with the remaining scrap being less than 5% or unclassified into a scrap category (such as alloyed steels). One specific challenge relevant to the scrap quality is the residual elements (or impurity elements) inherited from steel scrap such as Cu, Sn, Sb, Pb, Zn and Cr. This will become severe after repeated recycling via the EAF steelmaking route caused by the accumulated effect of elements such as Cu [38]. Such elements are noble compared with iron, and they will not be removed into slag or off-gas by oxidation or reduction during the EAF steelmaking, and consequently, their level in the metallic products could increase by 120% (e.g. Cu) without dilution in the raw materials.

**Table 2.** UK Scrap categories and expected residual quantities as derived from EU standards [45]

scrap category	UK Scrap ID	Cu %	Ni %	Sn %	Mo %	Cr %
0A – demo	0A	0.15	0.05	0.005	0.05	0.1
1 – thick old	1	0.25	0.05	0.01	0.05	0.15
2 – thin old	2	0.4	0.6	0.02	0.13	0.24
3B – fragmentized	3B	0.25	–	0.03	0.05	0.25
6A – cans + incinerated	6A	0.5	–	0.07	0.005	0.01
7A – turnings	7A	0.4	0.43	0.03	0.1	0.56
8A – manufacturing off-cuts	8A	0.1	0.02	0.01	0.01	0.07
9A – old cast iron + rail	9A	0.4	0.02	0.01	0.01	0.07
9D – brake discs and wheel drums	9D	0.4	0.02	0.01	0.01	0.07
12A – new cast iron	12A	0.1	0.02	0.01	0.01	0.07

Daehn *et al*. [46] estimated that the amount of copper in scrap will exceed the amount that can be tolerated across all products by 2050 unless methods for improved control are introduced. Efficiently removing residual copper from steel scrap has been extensively explored from high-density shredding, solid-state pre-treatment via sulphides to vacuum distillation of copper-containing liquid steel, which are all proved in laboratory- or pilot-scale trials but not in commercial circumstances [47]. For example, compared with conventional shredding, high-density shredding forces materials through reduced-size grid openings with extended time (and therefore at higher cost) and improves the separation of residual elements such as Cu and dirt from the steel scrap. The Cu level of the steel scrap after high-density shredding can be reduced to around 0.1% from 0.2 to 0.3% Cu in conventional shredding [47], which is at a level similar to the best practice of handpicking after conventional shredding. Recently, development for efficient control of scrap quality led by one of the current authors (Z Li) is by using AI-based tools (in combination with advanced analytic techniques) to improve scrap sorting during scrap processing and for scrap quality management in the scrap yard [48].

Another upstream attempt is to better disassemble the steel products and steel-containing products. Dynamic material flow analysis showed that only 7–8% of recovered car steel is recycled back into the automotive sector and the majority is used in buildings and civil engineering [49]. Without changing the current dismantling practice, steel scrap contamination by Cu will be exacerbated after switching to electric vehicles (EVs) as EVs have up to 3.5 times Cu usage compared with internal combustion vehicles. Extensive research has also been carried out by the authors to study the effects of residual elements on the downstream processabilities and steel product properties aiming at developing high residual elements-tolerant steels and changing the downstream processing parameters to manipulate the microstructure of the high residual elements containing steel without sacrificing the steel properties. This will be discussed in a later section with specific examples.

To efficiently control the residual elements levels in scrap-based EAF steelmaking, a holistic technological approach is required, as described above, from the EoL steel product disassembly, AI-aided scrap sorting and scrap management, scrap pre-treatment in the solid state, removal of residuals in liquid steel and increasing residual tolerance in the downstream process. However, if all the measures mentioned above cannot control the residual elements to an acceptable level for the steel grade manufactured, dilution with H-DRI is necessary. The percentage of the H-DRI used for dilution will depend on the balance of several factors such as the scrap quality, the steel grade made and costs.

Increased reuse and remanufacturing extend the lifetime of products and will reduce the demand for ‘new’ steel and therefore mitigate the increase in residual levels over time through recycling. However, it is currently inevitable that the residual element content in steels will increase in the future; therefore, it is important to consider some of the implications of this increase, which is demonstrated next with case studies.

### (a) Case studies on the effect of residuals

**Figure 4.**
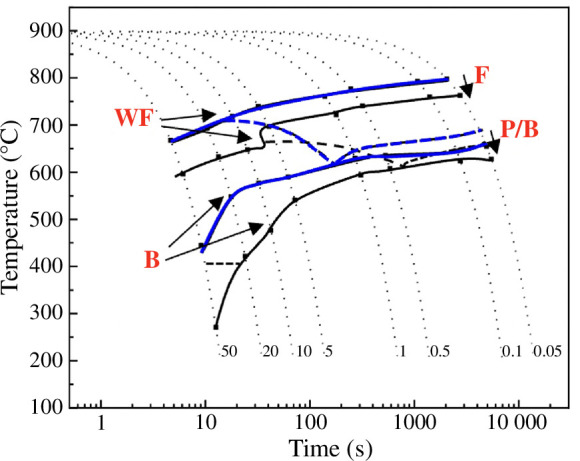
CCT diagram for a 0.17 wt% C steel (blue lines) and the same steel-containing residual elements (0.58 wt% Cu, 0.30 wt% Ni and 0.06 wt% Sn; black lines) showing the shift in the curves and increase in hardenability due to the residual elements. F: ferrite; WF: Widmanstatten ferrite; P: pearlite; B: bainite.

**Figure 5.**
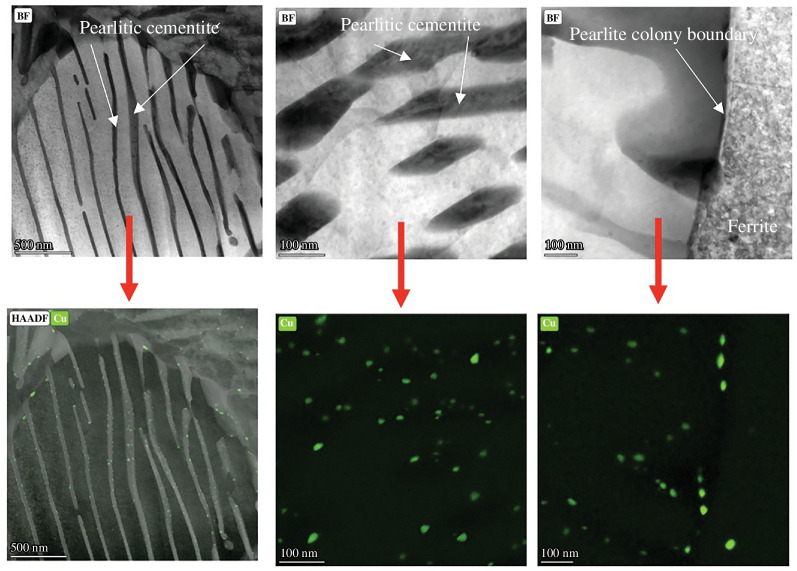
Transmission electron microscopy (TEM) images (upper row) and energy dispersive spectroscopy (EDS) images for Cu (lower row) of a 0.17 wt% steel containing 0.60 wt% Cu showing fine Cu precipitates on the pearlitic cementite lamellae and pearlite colony boundary. Note the lack of Cu precipitates in the ferrite region.

The consequences of increased levels of residual elements on steel processing and product properties need to be considered. High levels of elements such as Cu are not a problem for all steel grades, with some steels having deliberate additions of Cu (>1 wt%) to provide precipitate strengthening. These include grades such as high strength low alloy (HSLA−100) steel in the USA, which is used to give high strength and good bendability, allowing 20–30% less weight in products compared with using standard carbon steel [50]. At residual levels (<<1 wt%) in steel, Cu can still affect the strength, which could be an advantage in that the overall alloy composition can be made leaner to meet the same strength levels, giving a cost-saving, or higher strength grades can be produced for the same alloy content, provided other mechanical properties are not compromised. The effect of Cu, and other residual elements, on strength can be via affecting the transformation kinetics and hence final microstructure (unless process parameters are altered; figure 4), where residual elements increase the hardenability of the steel by shifting the continuous cooling transformation (CCT) curves to lower temperatures and longer times. This can be accommodated during steel processing by controlling the cooling rates after hot processing. Strength is also influenced by solid solution/precipitate strengthening, and it has been found that Cu precipitates generally form on interfaces and grain boundaries in the steel, which may limit the strengthening effect as these features already provide strengthening (figure 5).

**Figure 6.**
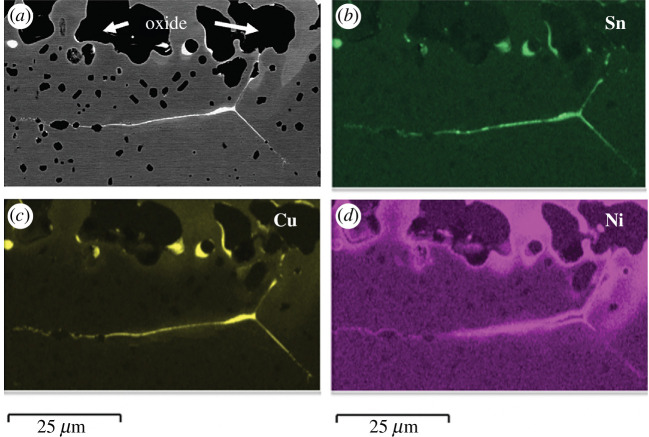
Backscattered SEM image (*a*) showing the enrichments of residual elements at the grain boundary with EDS maps of (*b*) Sn, (*c*) Cu and (*d*) Ni [43].

High residual levels have been associated with processing problems such as hot shortness, which is the tendency of a metal to fail in a brittle manner along grain boundaries when hot deformed. Hot shortness has been linked with the enrichment of residual elements at grain boundaries. Locally high concentrations of residual elements arise during the solidification of the liquid steel on casting because of micro-segregation, with residual elements partitioning from the solidifying steel into the remaining liquid. The equilibrium partition ratio is defined as the ratio between the concentration of the element in the solid divided by the concentration of the element in the liquid. Different elements show a different tendency to segregate with an equilibrium partition ratio for solidification as delta ferrite of 0.70 for Cu [51] and approximately 0.27 for Sn [52]. Therefore, both elements enrich in the last liquid to solidify. In addition to micro-segregation, further enrichment of residual elements can occur during oxidation at the oxide–metal interface due to the preferential oxidation of Fe, as well as local diffusion to interfaces and grain boundaries. When the local Cu (and Sn) level exceeds the solubility limit at high temperatures, then a liquid phase can form and penetrate along the grain boundaries weakening them. The presence of stress, in the form of thermal stresses during cooling, or mechanical stresses from straightening of continuous cast strands or hot deformation, can then cause cracking at the oxide–metal interface and particularly along the enriched grain boundaries. Examples of residual element enrichment along grain boundaries can be seen in figure 6 for a carbon (0.15 wt%) steel containing 0.6 wt% Cu, 0.15 wt% Ni and 0.06 wt% Sn that has been oxidized in air for 3 h at 1180°C, representative of a typical reheat schedule [53]. Mitigation measures against hot shortness include limiting the residual content in the steel, reducing oxidation enrichment via limiting reheating times/temperatures and controlling the reheat atmosphere, as well as limiting the mechanical stresses at high temperatures. In addition, increasing the Ni content in the steel has been shown to minimize hot shortness cracking, as it increases the solubility of Cu in the steel minimizing the formation of the liquid Cu rich phase that penetrates and embrittles grain boundaries.

**Figure 7.**
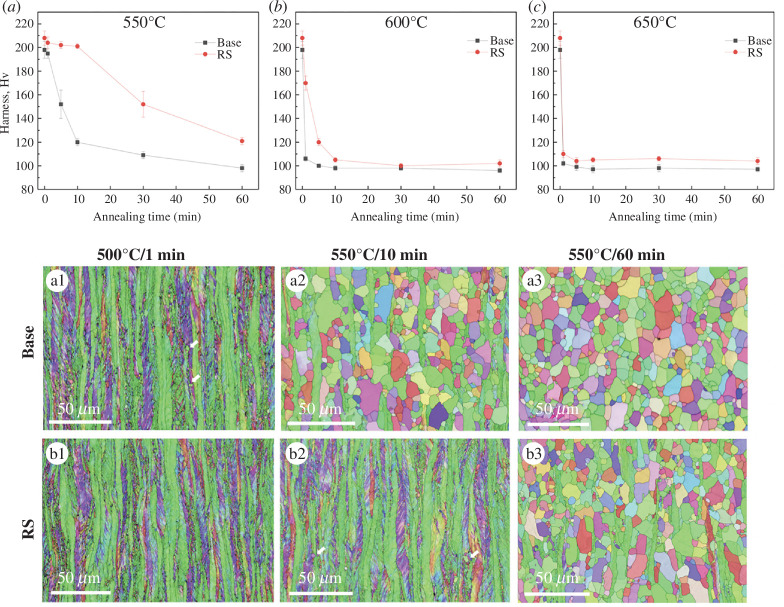
Recrystallization progress (indicated by the reduction in hardness) and microstructures for a low-carbon (0.02 wt%) steel (base) and the base composition with residual elements (RS): 0.15 wt% Cu, 0.16 wt% Ni and 0.04 wt% Sn, cold rolled to 73% reduction and annealed at (*a*) 550°C, (*b*) 600°C and (*c*) 650°C. Microstructures in the Base (*a1, a2, a3*) and RS (*b1, b2, b3*) samples after annealing at 550°C for 1, 10 and 60 min, with strain-free small grains arrowed indicating the onset of recrystallization [44].

Residual elements can affect other aspects of microstructure development during processing. An example is the process of recrystallization, where deformed grains are replaced with new, strain-free, grains that nucleate and grow within the strained grains. Recrystallization occurs during hot deformation and during heat treatment (annealing) after cold rolling and is an important process for controlling the final grain size and texture in the steel, which influences strength, formability and toughness. The recrystallization kinetics are affected by numerous factors, including strain, grain size, temperature and the steel composition with elements such as Nb having a significant effect on slowing the recrystallization process. It has been shown that residual elements can also affect the recrystallization kinetics (figure 7), with recrystallization being slowed by the presence of Cu and Sn [54]. The final microstructure (grain size and texture) was unaffected for this grade of steel once full recrystallization was achieved. The implications for this steel are that processing parameters may need to be altered when producing higher residual content steels, but the final microstructures and properties may be less affected.

In terms of downstream processing by steel customers and final product performance, then the effect of residual elements on, e.g. weldability and corrosion, should be considered. Upper compositional limits, usually defined by a carbon equivalent equation, are considered for welding, which reflects the tendency for the formation of martensite (a brittle phase). A number of different carbon equivalent equations were developed for different grades of steel. A commonly used measure for modern low-carbon steels is the P_CM_ value, developed in Japan, calculated using equation (4.1) [55]:


(4.1) $$P_{CM}=C+Si/30+(Mn+Cu+Cr)/20+Ni/60+Mo/15+V/10+5B.$$

As can be seen in equation (4.1), the effect of Cu is taken into account for weldability; therefore, if Cu residual levels increase in steel, there is potential for issues to arise if the composition is already close to the weldability limit. Mitigation would be altering the base composition levels, for example, lowering the C content with any strength deficit to be offset by Cu solid solution/precipitation strengthening. Alternatives would be via changes to the welding parameters, for example using a lower heat input or preheating the steel. Sn is not included in the carbon equivalent equations, but the residual levels of Sn in steel are generally much lower than Cu and therefore might be considered to have a secondary effect. Researchers have considered the effect of Sn and at higher levels significant detrimental effects on the base steel mechanical properties, including through-thickness properties, which can be related to lamellar tearing on welding, have been observed [56]. Cu is generally considered to be beneficial to corrosion resistance with deliberate Cu additions being made to weathering steels and reports of reduced corrosion levels when Cu is present in the steel [57,58]. Therefore, changes in steel chemistry due to increased scrap use can have both negative and positive effects on processing and final properties, requiring careful consideration to determine what modifications in composition, processing parameters and product performance may arise.

## 5. Summary

The steel sector is moving to lower CO_2_ emission manufacturing with an increase in EAF production coupled with initiatives to increase the reuse and remanufacturing of steel-intensive products. Opportunities and challenges arise associated with these changes. Improved materials tracking (materials passports) is required to establish EoL quality, needing in-service monitoring to establish damage accumulation, and to inform decisions on whether components can be reused, remanufactured or need to be recycled. Design for disassembly becomes important to support these approaches along with new supply chains and business models. Recycling will become even more necessary as steel manufacturers increase EAF use and increase scrap input into the BF-BOF process, with improved scrap sorting and quality control being essential to supply low residual content scrap to support the production of all steel grades. The accumulation of residual elements, such as copper and tin, from recycled steel scrap can provide benefits, for example strengthening the steel, allowing a lower amount of intentional alloying additions to be used, or cause disadvantages, such as limitations in scrap use and process parameter changes to avoid hot shortness or undesirable microstructures. Future considerations could include collaborations between steel producers and customers to redesign products for improved disassembly, reuse, remanufacture and recycling, including simplification of the number of alloys used in a product, such as the ‘Unisteel’ concept proposed recently for automotive applications [59]. It should be noted that while reuse and remanufacturing will reduce CO_2_ emissions in the steel sector, the transition to low CO_2_ emission technologies for primary steel manufacture (such as EAF) is key in moving towards net zero. However, since the global supply of scrap steel is limited and geographically focused in certain countries (mostly Europe and Northern America), there is still a need for development of low CO_2_ emission technologies (such as H-DRI, use of alternative fuels (biomass)) to support BF-BOF production of steel.

## Data Availability

Additional data that are relevant to figure 3 have been uploaded as supplementary material [60].
